# Absolute Quantification of Donor-Derived Cell-Free DNA in Pediatric and Adult Patients After Heart Transplantation: A Prospective Study

**DOI:** 10.3389/ti.2023.11260

**Published:** 2023-10-30

**Authors:** Jens Böhmer, Carina Wasslavik, Daniel Andersson, Anders Ståhlberg, Marianne Jonsson, Håkan Wåhlander, Kristjan Karason, Jan Sunnegårdh, Staffan Nilsson, Julia Asp, Göran Dellgren, Anne Ricksten

**Affiliations:** ^1^ Sahlgrenska University Hospital, Gothenburg, Sweden; ^2^ Sahlgrenska Academy, University of Gothenburg, Gothenburg, Sweden; ^3^ Sahlgrenska Cancer Center, University of Gothenburg, Gothenburg, Sweden; ^4^ The Wallenberg Centre for Molecular and Translational Medicine, University of Gothenburg, Gothenburg, Sweden; ^5^ Transplant Institute, Sahlgrenska University Hospital, Gothenburg, Sweden; ^6^ Laboratory Medicine, University of Gothenburg, Gothenburg, Sweden

**Keywords:** heart transplantation, rejection, prospective follow-up, cell free DNA, surveillance

## Abstract

In this prospective study we investigated a cohort after heart transplantation with a novel PCR-based approach with focus on treated rejection. Blood samples were collected coincidentally to biopsies, and both absolute levels of dd-cfDNA and donor fraction were reported using digital PCR. 52 patients (11 children and 41 adults) were enrolled (NCT03477383, clinicaltrials.gov), and 557 plasma samples were analyzed. 13 treated rejection episodes >14 days after transplantation were observed in 7 patients. Donor fraction showed a median of 0.08% in the cohort and was significantly elevated during rejection (median 0.19%, *p* < 0.0001), using a cut-off of 0.1%, the sensitivity/specificity were 92%/56% (AUC ROC-curve: 0.78). Absolute levels of dd-cfDNA showed a median of 8.8 copies/mL and were significantly elevated during rejection (median 23, *p* = 0.0001). Using a cut-off of 7.5 copies/mL, the sensitivity/specificity were 92%/43% for donor fraction (AUC ROC-curve: 0.75). The results support the feasibility of this approach in analyzing dd-cfDNA after heart transplantation. The obtained values are well aligned with results from other trials. The possibility to quantify absolute levels adds important value to the differentiation between ongoing graft damage and quiescent situations.

## Graphical Abstract

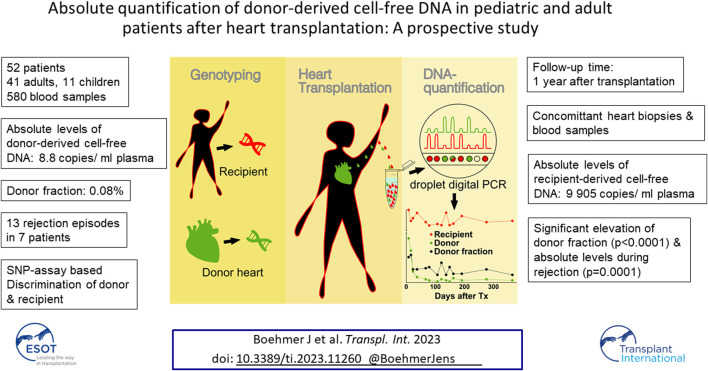

## Introduction

Patients with advanced heart failure can undergo heart transplantation (HTx) as a definite treatment option. Acute and chronic rejection are major factors contributing to limited survival after HTx [1–4]. The diagnosis of rejection requires surveillance with endomyocardial biopsies and histopathological studies [5–7], which show a high interobserver-variability [8]. A less invasive and less costly approach by reliable biomarkers is thus desirable, ideally with the possibility of timely diagnosis.

Cell-free DNA (cfDNA) is released into the bloodstream after cell apoptosis or necrosis and is mostly of hematopoietic origin [9–13]. Levels of cfDNA show a large inter- and intraindividual variability and vary between 0 and 5 ng/mL to >1,000 ng/mL; elevations are seen during both physiological and pathological situations (exercise, cancer, sepsis etc.) [14, 15]. Donor-derived cell-free DNA (dd-cfDNA) can be differentiated from recipient-derived cell-free DNA (rd-cfDNA) and has been correlated to rejection [16–19]. The quota of dd-cfDNA to total cfDNA, termed the donor fraction (DF), has been used for graft surveillance as the sole reported measure. Recent studies, however, have advertised the addition of absolute levels of dd-cfDNA for this purpose [20–22] considering not only DF, but also the high variability of rd-cfDNA. A steady state of 0.1% DF has been a consistent finding after HTx [18, 19, 23–25], which is the lowest among all solid organ transplantations [26–28]. The low abundance makes the use of highly sensitive quantification techniques mandatory. Digital PCR (dPCR) offers an absolute quantification of cfDNA in combination with quick turn-around time and high sensitivity [29–32].

In this non-interventional prospective cohort study, we describe a novel approach for the analysis of dd-cfDNA after HTx, using a technique including dPCR, and SNP (single nucleotide polymorphism) genotypes with target-specific preamplification. We aimed to establish a standardized protocol and then use the technique on patients after HTx. The primary objective of the study was to show if the use of donor fraction DF in HTx-patients can differentiate rejection from the absence of rejection, compared to the results of endomyocardial biopsies.

Secondary goals were: the investigation of absolute levels of dd-cfDNA with treated rejection events, differences between early and later samples after HTx as well as differences between female versus male recipients and adults versus children, respectively.

## Patients and Methods

### Patient Recruitment

Patients were recruited from Sahlgrenska University Hospital, Gothenburg, Sweden as a part of the prospective BIODRAFT-trial (NCT03477383). All patients or caregivers provided informed consent and the study was approved by the institutional review board (no 014-16). Patients were eligible if they underwent HTx between 2016 and 2018. Blood samples were drawn coincidental to endomyocardial biopsies (EMB) during the first year after HTx. Access to donor blood samples was granted. Clinical data were extracted from medical records.

### Sample Preparation

Blood was collected immediately before catheterization in 10 mL Cell-Free DNA^®^ BCT (Streck, La Vista NE, USA). The samples were agitated for 10 s, shipped, and stored at room temperature for no longer than 7 days before plasma isolation. Plasma was separated from cells by centrifugation at 2,000 g for 15 min. The plasma fraction was transferred to a new tube followed by a second centrifugation at 16,000 g for 10 min. Centrifugations were performed at 20°C. The plasma was transferred to a collection tube and frozen at −80°C. Genomic DNA was prepared from the leukocyte fraction of the blood samples using the DNeasy Blood & Tissue Kit (Qiagen GmbH, Hilden, Germany). cfDNA was extracted using the QIAamp^®^ Circulating Nucleic Acid Kit (Qiagen) on the QIAvac 24 Plus vacuum manifold (Qiagen). cfDNA was eluted in 20 μL AVE buffer per ml plasma. Samples were stored at −20°C until analysis. Concentrations of cfDNA were quantified with the Qubit^®^ 3.0 Fluorometer (Thermo Fisher Scientific, Waltham MA, USA), fragment sizes were analyzed with the 4200 TapeStation (Agilent technologies, Santa Clara CA, USA).

### Discrimination of Donor and Recipient

35 previously published SNP assays [29] were selected for this study. For detection of the Y-chromosome the Human Y-Chromosome Specific Assay (TATAA Biocenter, Gothenburg, Sweden) targeting the TSPY1 gene was used. Probe and primer sets (Integrated DNA Technologies Inc., Carolville, IA, USA) were designed using HEX (Hexachlorofluorescin) and FAM (Carboxyfluorescin).

### Genotyping of Recipient and Donor

Genomic DNA extracted from white blood cells was used. The donor was investigated with respect to the homozygous alleles found in the recipient. In sex mismatched HTx (female recipient, male donor), the Y-chromosome was used.

### Target-Specific Preamplification

Preamplification using pooled primers for all 35 SNP and the Y-chromosome was conducted on cfDNA corresponding to 2 mL of patient plasma. 40 μL cfDNA, 45 μL Q5 Hot Start High-Fidelity 2x Master Mix (New England BioLabs, Ipswich, MA, USA), 3.6 μL primerpool (0.04 μmol) and 1.4 μL water were used in a total volume of 90 μL. Amplification was applied on a T100 Thermal Cycler (Bio-Rad, Hercules, CA, USA): 98°C for 3 min, followed by 10 cycles (98°C for 20 s, 63°C for 3 min and 72°C for 30 s). After the final extended (10 min) elongation step, the samples were stored at −20°C until analysis.

### dPCR on Non-Amplified cfDNA

dPCR on non-amplified cfDNA was conducted on all patient samples using a targeted SNP assay. 10 μL of eluted patient cfDNA, corresponding to 0.5 mL of blood plasma, were used with 11 μL ddPCR Supermix for Probes (No dUTP) (Bio-Rad), 0.5 μL primer/probe-mix (900 nmol of each primer and 250 nmol of each probe) and 0.5 μL water in a total volume of 22 µL. Negative control with a no template control (NTC) with water was included as well as a positive control with a sample of genomic DNA (gDNA). Amplification was applied on a T100 Thermal Cycler (Bio-Rad): 95°C for 10 min, followed by 40 cycles (95°C for 30 s, 59°C/61°C for 1 min), 98°C for 10 min. Analysis was performed using the QX200 AutoDG Droplet Digital PCR System (Bio-Rad). Using negative and positive controls for cluster detection, manual fluorescent thresholds were placed. Analysis was conducted using QuantaSoft Analysis Pro v1.0 (Bio-Rad) to calculate absolute droplet counts as well as target DNA-concentration for rd-cfDNA. Target concentrations were expressed as copies per ml plasma.

### dPCR on Target Specific Preamplified cfDNA (PA-dPCR)

dPCR on the preamplified cfDNA (PA-dPCR) was conducted using dilutions, based on the target concentrations of non-amplified cfDNA. All identified SNP were used. 10µL amplified cfDNA was diluted in purified water to acquire the desired concentration. All experiments were conducted as triplets, according to the protocol in 2.6. Target concentrations were expressed as copies per µl PCR-reaction, DF was calculated as dd-cfDNA/(rd-cfDNA + dd-cfDNA).

### Calculation of cfDNA (cp/mL Plasma)

The initial dPCR is conducted on known concentrations directly corresponding to the amounts of isolated plasma (3 mL plasma is eluted in 60 μL, 4 mL plasma in 80 μL), see Supplementary Figure S1). This allows for the determination of absolute copy numbers for the recipient (cp/mL plasma). Using the donor fraction from the PA-PCR, by multiplying with absolute copy numbers for the recipient, total copy number for the donor is calculated (cp/mL plasma).

### Determination of Assay Performances

The efficiency of target-specific preamplification was determined using a cfDNA control from normal donor plasma, in the range of 0.5–32 ng. Preamplification was performed in 30 μL reactions, using Q5 Hot Start High-Fidelity 1x Master Mix, 40 nM of each primer and template cfDNA at seven different concentrations (32, 16, 8, 4, 2, 1, and 0.5 ng/μL) in triplicate. The same amplification protocol was used as above. After the final extended (10 min) elongation step, the samples were immediately frozen on dry ice, slowly thawed on ice, diluted 1:20 in 1x TE buffer (ThermoFisher Scientific, Waltham, MA, USA) and stored at −20°C until analysis. qPCR (real-time quantitative PCR) was performed in 10 μL reactions utilizing 1x TATAA SYBR GrandMaster Mix (TATAA Biocenter) with 400 nM of each primer (Integrated DNA Technologies) and 2 μL diluted preamplification product as template. qPCR was performed in triplicates using the CFX384 Touch Real-Time PCR Detection System (Bio-Rad): 95°C for 10 min, followed by 50 cycles of amplification (95°C for 15 s and 60°C for 1 min). Melting curve analysis was performed in the range of 65°C to 95°C, 0.5°C per 5 s increments. Cycles of quantification (Cq) values were determined by the second derivative maximum method. Limit of blank (LOB), limit of detection (LOD) and limit of quantification (LOQ) were defined according to Armbruster et al [33] and determined as published by our group [34]: LOB 0.016% DF, LOD 0.055% DF, with LOQ = LOD.

### Statistics

Quantification of cfDNA in the dPCR-experiments is embedded in the software Quantasoft Pro (Bio-Rad) [35]. Continuous data are reported as means with standard deviation (SD) or as medians with interquartile range (IQR, q1-q3). Range is also reported when appropriate. In the boxplots, the horizontal line represents the median value, the top and bottom of each box show the upper and lower limit of the IQR, and the whiskers represent the range. Receiver-operating characteristic (ROC) curves were used to assess the sensitivity and specificity of DF and dd-cfDNA to predict treated rejection. Correlations (Pearson and Spearman) report the correlation coefficient r, CI_95_ of r and R^2^. Early (7–14 days) and late (>14 days) samples were compared using a linear mixed effects (LME) model. Other groups were also compared using a LME model, with adjustment for days from transplantation. A two-tailed *p*-value of <0.05 was considered statistically significant. The LME models were computed using R Statistical Software (v4.3.1) [36]. All other statistical analysis was performed using the GraphPad Prism Software (GraphPad Software Inc., version 10.0.2, GraphPad Software, Boston, Massachusetts USA).

## Results

### Study Population

The 52 patients generated 580 venous samples during their 1 year follow-up. 23 samples were excluded (hemolytic sample, too little plasma yield, high technical error rate, too few droplets generated). The population consisted of 41 adults and 11 children who were aged 1–68 years, (median 52.5), 69% of patients were male, and the median BMI was 24.9. The indication for transplant was dilated cardiomyopathy in 58%, and ventricular assist device was used in 29%. Median donor age was 49.5 years, median donor BMI was 23.8, median donor heart ischemic time was 183.5 min. More detailed patient and donor specifications can be seen in the Supplementary Tables S1, S2. Median time to first biopsy was 10.5 days (IQR 9–12). Blood samples taken during the first 14 days after HTx (*n* = 48) were analyzed by dPCR but excluded from general statistical analysis except for when otherwise stated.

### Study Protocol and General Results

The detailed workflow and calculation of cfDNA-levels from recipient, donor and DF can be seen in Supplementary Figure S1. After sample collection, cfDNA was extracted within a time frame of 7 days [37]. cfDNA-analysis was conducted on bundled samples after the patients had left the study. Results were available with 48 h. A mean of 4 mL plasma was obtained from each sample (SD 0.57, range 1.25–5.70). The median for the fluorometrically determined cfDNA-concentration was 34.20 ng/mL plasma (range 5.16–2,856, IQR 20.45–57.60). The 509 samples showed a median of 9,905 copies/mL plasma rd-cfDNA concentration (range 1,245-219,754, IQR 5,137-17,596), the median for dd-cfDNA was 9.31 copies/mL plasma (range 0.43–348.5, IQR 5.06–21.71, mean 18.9). Donor fraction showed a median of 0.09% (range 0.003–3.34, IQR 0.05–0.21). See Supplementary Figure S2.

### Rejection and Levels of cfDNA

**FIGURE 1:**
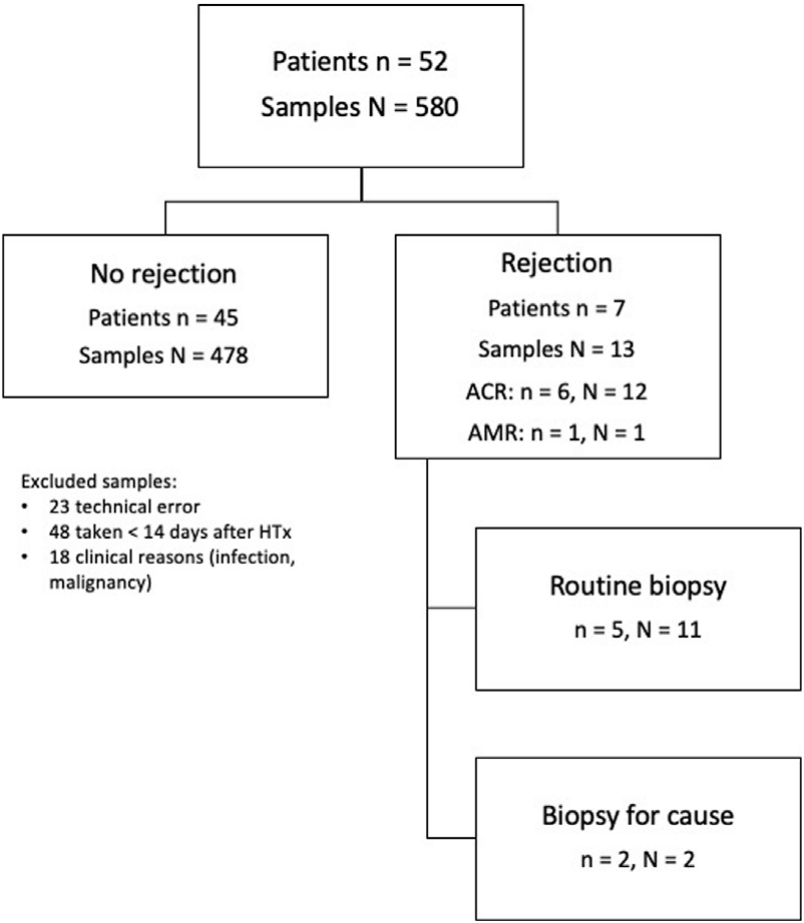
Study flow chart. ACR, acute cellular rejection; AMR, antibody-mediated rejection; HTx, heart transplantation.

**FIGURE 2:**
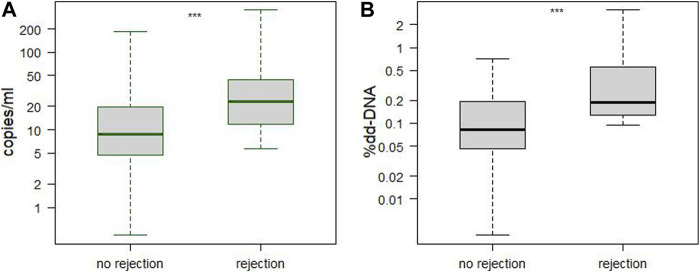
Comparison of samples taken during episodes of acute rejection versus no rejection. Panel **(A)** shows absolute levels of dd-cfDNA in copies/mL, Panel **(B)** shows DF in percent. Dd-cfDNA was elevated in rejection (median 23.0, IQR 10.6–49.8) versus no rejection (median 8.9, IQR 4.7–20.3), *p* = 0.0001. DF was also elevated in rejection (median 0.19, IQR 0.13–0.56) versus no rejection (median 0.08, IQR 0.05–0.19), *p* < 0.0001. No rejection: N = 45 patients, *n* = 479 samples; rejection: N = 7, *n* = 13. cfDNA, cell-free DNA; dd-cfDNA, donor-derived cfDNA; DF, donor fraction; IQR, interquartile range; rd-cfDNA, recipient-derived cfDNA.

Of the 557 samples, 48 samples were excluded as early samples (<15 days) and 18 due to reasons that impaired interpretation (malignancy, severe infections). This resulted in 491 samples that were suitable for evaluation. Acute reaction was seen in 13 biopsy-matched samples from 7 patients, see Figure 1. One sample showed rd-cfDNA levels of 220,000 copies/mL, >20 times the median (see Study Protocol and General Results), falsely lowering the DF, and was thus excluded from analysis. Median levels were significantly higher during rejection episodes: absolute levels of dd-cfDNA showed a median of 23 copies/mL (IQR 10.6–49.8) during rejection compared to 8.8 (IQR 4.7–19.8) during quiescence (*p* = 0.0001). Using a cut-off of 7.5 copies/mL, sensitivity was 92% and specificity was 43% (AUC 0.75; 95% CI 0.63–0.87, PPV = 0.04, NPV = 0.99). DF was also elevated in rejection, with a median level was 0.19 (IQR 0.13–0.56) compared to 0.08 (IQR 0.05–0.19) during quiescence (*p* < 0.0001). Using a cut-off of 0.1%, sensitivity was 92% and specificity was 56% (AUC 0.78, 95% CI 0.68–0.88, PPV = 0.05, NPV = 0.99). See Figure 2 and Supplementary Figure S7.

### Group Comparisons

Early samples (day 7–14, *n* = 48) were compared to later samples (day 15–400, *n* = 509). The early samples showed significantly higher levels (*p* < 0.0001) for both rd-cfDNA (median 18,135 versus 9,905 copies/mL), dd-cfDNA (median 48.7 versus 9.3 copies/mL) and DF (median 0.31% versus 0.09%), see Supplementary Figure S3.

Samples from adults (*n* = 475) were compared to samples from children (*n* = 82), including early samples. Levels of rd-cfDNA did not differ significantly (adults median 11,091 copies/mL versus 7,177, *p* = 0.053). Levels of dd-cfDNA did not differ significantly (adults median 11.0 copies/mL versus children 9.8, *p* = 0.99). DF was significantly lower in adults (median 0.09 versus 0.13, *p* = 0.03), see Supplementary Figure S4.

Female patient samples (*n* = 171) were compared to samples from male patients (*n* = 386). As compared with females, males had significantly lower levels of rd-cfDNA (median 8,888 copies/mL versus 15,943, *p* = 0.0004). There were no significant differences for dd-cfDNA (males median 9.8 copies/mL versus 13.2, *p* = 0.13) and DF (males median 0.12% versus 0.09%, *p* = 0.08), see Supplementary Figure S5.

### Correlations and Validation of Assay Performance

The rd-cfDNA results of the initial dPCR (copies/mL) and the PA-dPCR (copies/µL) were compared including results from samples taken during the first 14 days after HTx (*n* = 557). The results showed a very high correlation (Pearson r = 0.97, CI_95_ (0.97; 0.98), R^2^ = 0.95, *p* < 0.0001; Spearman r = 0.95, CI_95_ (0.94; 0.96), *p* < 0.0001). The levels of rd-cfDNA from PA-dPCR were also correlated with the fluoroscopic measurements of DNA-concentration in the initial plasma samples using Qubit. The results showed a very high correlation (Pearson r = 0.95, CI_95_ (0.94; 0.95), R^2^ = 0.90, *p* < 0.0001; Spearman r = 0.93, CI_95_ (0.92; 0.94), *p* < 0.0001).

The efficiency of preamplification was determined using a cfDNA standard and qPCR to monitor individual SNP assays as previously published by our group (XX). The qPCR profiles for all SNP assays including the Y-chromosome are seen in Supplementary Figure S6. No changes in allelic distribution for the SNP assays could be detected within the range of cfDNA concentrations.

### Patient Examples

**FIGURE 3:**
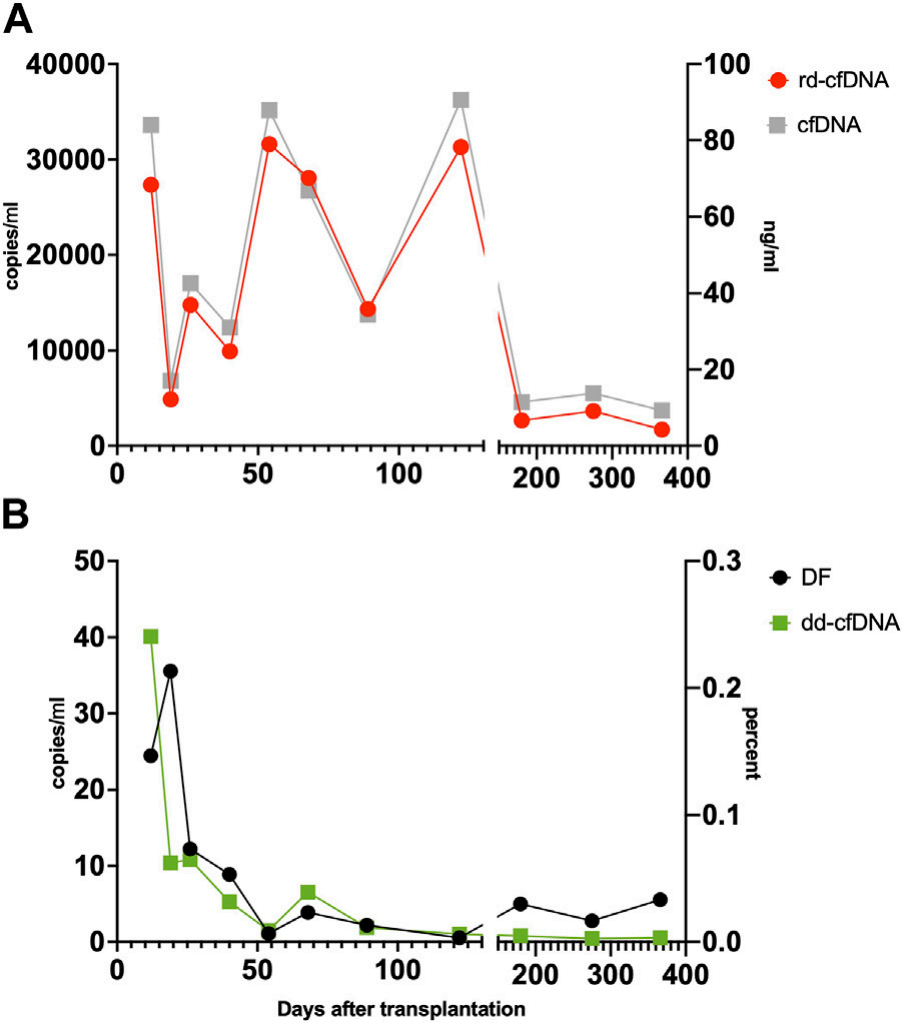
Patient 1. Time course of rd-cfDNA and cfDNA-concentration (Panel **(A)**) as well as dd-cfDNA and DF (Panel **(B)**). rd-cfDNA and dd-cfDNA results are displayed on the left Y-axis, respectively, expressed in copies/mL. DF is expressed in percent, on the right lower Y-axis. Results of rd-cfDNA, dd-cfDNA and DF are based on three SNP [16, 21, 25] displayed as means. Results of cfDNA are the fluoroscopic measurements of patient plasma (Qubit), expressed in ng/mL on the right upper Y-axis. The time course of this rejection-free patient shows large variation of rd-cfDNA without clinical correlates. Dd-cfDNA declines after HTx and remains low. The quick fall of rd-cfDNA leads to a rise in DF in the second sample, despite absence of rejection. There is a very close relation between the fluoroscopically determined DNA-concentration and the results for rd-cfDNA as determined by dPCR. cfDNA, cell-free DNA; dd-cfDNA, donor-derived cfDNA; dPCR, digital PCR; DF, donor fraction; rd-cfDNA, recipient-derived cfDNA.

Patient 1: A 27 year-old patient underwent uneventful HTx. The clinical course was unremarkable except for suspected bacterial infection on day 3 and day 20 as well as Influenza A infection on day 270. A total of 11 scheduled endomyocardial biopsies were obtained, none of which showed signs of rejection warranting treatment. The results are shown in Figure 3.

**FIGURE 4:**
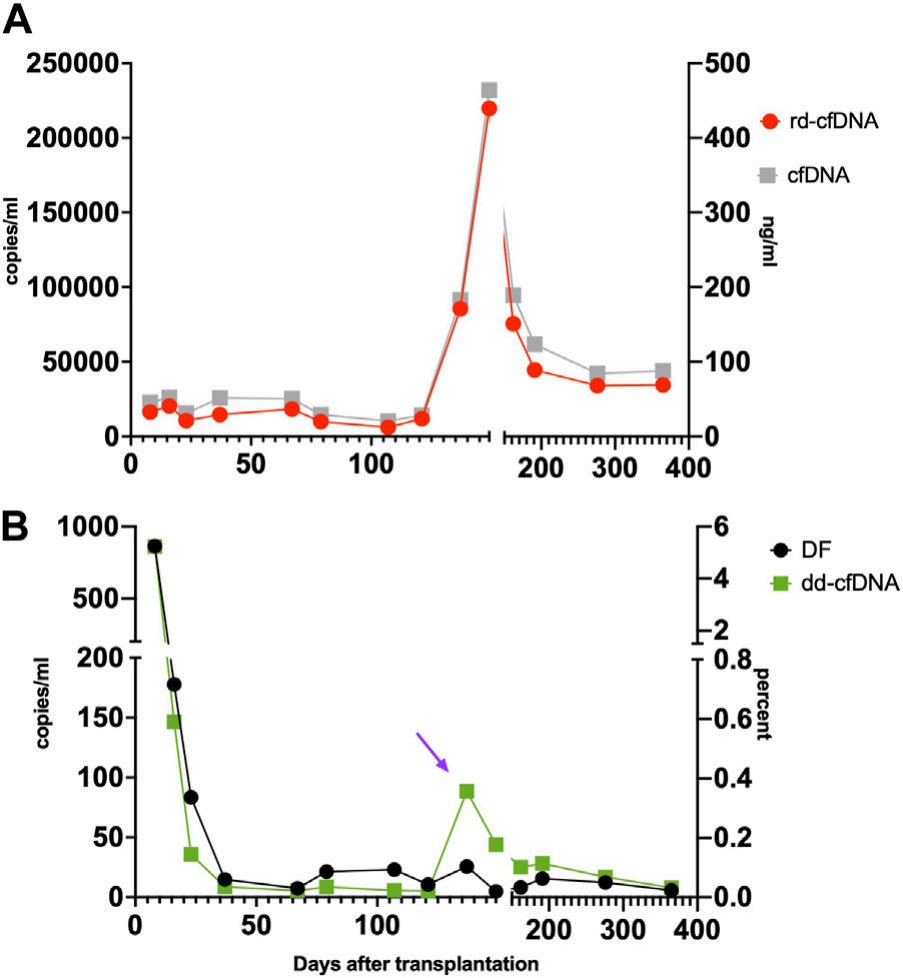
Patient 2. Time course of rd-cfDNA and cfDNA-concentration (Panel **(A)**) as well as dd-cfDNA and DF (Panel **(B)**). The time point of a treated rejection (IHLT-2R) as warranted by the biopsy is marked with a purple arrow. rd-cfDNA and dd-cfDNA results are displayed on the left Y-axis, respectively, expressed in copies/mL. DF is expressed in percent, on the right lower Y-axis. Results of rd-cfDNA, dd-cfDNA and DF are based on three SNP [14, 16, 25], displayed as means. Results of cfDNA are the fluoroscopic measurements of patient plasma, expressed in ng/mL on the right upper Y-axis. The rejection is reflected by a marked rise in dd-cfDNA, as well as treatment response by a decline. This is, however, masked by the coincidental rise in rd-cfDNA, which thus prevents DF to rise significantly. cfDNA, cell-free DNA; dd-cfDNA, donor-derived cfDNA; DF, donor fraction; rd-cfDNA, recipient-derived cfDNA.

Patient 2: A 28 year-old patient underwent uneventful HTx. Scheduled routine biopsy on day 137 showed a 3R rejection and treatment was initiated. Further biopsies revealed resolution of the rejection. Infection episodes occurred on day 4 and day 94 (respiratory infections), day 125 (infection with enterovirus) and day 164 (urinary tract infection). The results are shown in Figure 4.

## Discussion

In the present study, a median DF levels of 0.09% is well aligned with that observed in previous studies in the field. Special attention should be given to the fact that the results are comparable despite the different technical approaches such as sequencing [24], massive multiplexed PCR [19], and PCR with a preselected SNP-set [38, 39], as used in our study. As has been shown before, DF is significantly higher in samples taken coincidental with biopsies showing acute rejection. Even absolute values of dd-cfDNA are significantly higher during rejection, a result shown in kidney transplantation [20–22], and in HTx recipients as shown by Kim et al. [25], even though Kim et al. did not reveal how absolute levels were quantified. It must be noted that absolute levels of cfDNA in our cohort showed very high intra- and interpatient variability (up to the factor 180), and the influence of this on DF is well illustrated in Figure 3: On the second sample, DF rises, and rejection can be suspected. This can, however, be explained by the kinetics of the rapid decline of rd-cfDNA compared to the modest decline in dd-cfDNA. Clinically, the peaks seen in some patients could be correlated to infection and bleeding, but sometimes no obvious reason could be found. Also, the differences seen between male and female patients regarding rd-cfDNA levels remain to be explained. Similarly, large variations in rd-cfDNA after transplantation have been noted by others [21]. Interestingly, total levels of dd-cfDNA seem to correlate as well between the different studies, and also between the different organs: Even though no range or IQR is given, Kim et al. propose a threshold of 13 copies/mL as being superior to DF in diagnosing rejection, which can be compared to the median of 9.3 copies/mL in our study. In stable kidney transplants, the dd-cfDNA levels showed a median of 25 copies/mL [20], which, however, must be viewed on the background of the higher median for DF (0.29%). Biologically, this similarity seems logical, given the similar organ sizes of heart and kidney (both around 300 g in males) and reflecting the higher cell-turn around in kidneys compared to the heart.

The technical robustness of our approach is supported by two findings: the comparison of our results to other studies and the very high correlation when comparing total cfDNA-concentration measured by Qubit with rd-cfDNA results from PA-dPCR. Even the very high correlation between rd-cfDNA levels from the first and second dPCR supports this assumption.

In our opinion, technical approaches solely reporting DF may be unreliable as variations in rd-cfDNA are not being accounted for. For example, an increase in dd-cfDNA, suggesting acute organ rejection, may not be detected if rd-cfDNA is simultaneously increased. This is clearly seen in Figure 4, where the high levels of rd-cfDNA mask the ongoing rejection if only focusing on DF. Contrary to just delivering DF, the method described by our group allows to separately monitor rd- and dd-cfDNA. This approach has been postulated in kidney transplantation with promising results [20, 21]. In a recent review [40], the authors stressed the advantages of quantifying absolute levels of dd-cfDNA to overcome the large variability of rd-cfDNA.

The use of target-specific preamplification enables the repeated analysis of multiple targets and has been thoroughly discussed by Jackson and Andersson [41, 42]. Quantifying DNA always introduces the problem that assays can have different efficiencies. Using several, averaged assays is one way of minimizing this technical challenge. The different and complex technical approaches are one of the reasons why a systematic review on the use of cfDNA after organ transplantation [28], initially planned to perform a diagnostic test accuracy (DTA) meta-analysis, failed to do so.

In conclusion, we established a robust and fast method to quantify cell-free DNA as an indicator of rejection in cardiac recipients, which is less invasive and less costly than endomyocardial biopsy. The results from 52 patients, for whom DF was measured repeatedly, are in concordance with previous studies. Our method also allows for the measurement of cfDNA from the recipient and the donor, separately, providing more information than DF alone. Thus, our technique can be a promising tool for rejection-surveillance after HTx. Its usefulness will be examined by the BIODRAFT-trial (NCT03477383) comparing cfDNA-levels to clinical data of the patient cohort.

## Data Availability

The original contributions presented in the study are included in the article/Supplementary Material, further inquiries can be directed to the corresponding author.

## Abbreviations

cfDNA, cell-free; DNAdd, donor-derived; DF, donor fraction; dPCR, digital PCR; gDNA, genomic DNA; HTx, heart transplantation; NGS, next generation sequencing; NTC, no template control; PA, preamplification; PCR, polymerase chain reaction; qPCR, quantitative real-time; PCR rd, recipient-derived; SNP, single nucleotide polymorphism.
