# Supplementary material for: Absolute Quantification of Donor-Derived Cell-Free DNA in Pediatric and Adult Patients After Heart Transplantation: A Prospective Study
Source: Transpl Int. 2023 Oct 30;36:11260. doi: 10.3389/ti.2023.11260 (PMC10641041; doi:10.3389/ti.2023.11260)
Supplement: Supplementary file 1 [file DataSheet2.docx]

Supplemental tables

Supplemental table 1: Clinical characteristics of the patients

| All patients | 52 |
| --- | --- |
| Female | 16 (31%) |
| Age (years) | mean 43.3, median 52.5, range 1-68 |
| Body surface area (BSA) | mean 1.80, median 1.96, range 0.39-2.55 |
| BMI (kg/m^2^) | mean 25.05, median 24.92, range 13.97-35.11 |
| Indication for transplant |  |
| Dilated cardiomyopathy | 30 (58%) |
| Ischemic cardiomyopathy | 6 (23%) |
| Other cardiomyopathy | 7 (13%) |
| CHD: biventricular physiology | 5 (10%) |
| CHD: univentricular physiology | 3 (6%) |
| Retransplant | 1 (2%) |
| Kidney function pre-transplant |  |
| GFR ≥ 90 ml/min/1,73m^2^ | 9 (17%) |
| GFR 61-89 ml/min/1,73m^2^ | 16 (31%) |
| GFR 31-60 ml/min/1,73m^2^ | 25 (48%) |
| GFR 15-30 ml/min/1,73m^2^ | 2 (4%) |
| VAD use | 15 (29%) |
|  |  |
|  |  |
| Children | 11 |
| Female | 4 (36%) |
| Age (years) | mean 9.5, median 11, range 1-14 |
| Body surface area (BSA) | mean 1.00, median 1.00, range 0.39-1.31 |
| BMI (kg/m^2^) | mean 17.44, median 16.23, range 13.97-27.65 |
| Indication for transplant |  |
| Dilated cardiomyopathy | 6 (55%) |
| Ischemic cardiomyopathy | 0 (0%) |
| Other cardiomyopathy | 1 (9%) |
| CHD: biventricular physiology | 1 (9%) |
| CHD: univentricular physiology | 3 (27%) |
| Retransplant | 0 (0%) |
| Kidney function pre-transplant |  |
| GFR ≥ 90 ml/min/1,73m^2^ | 5 (45%) |
| GFR 61-89 ml/min/1,73m^2^ | 5 (45%) |
| GFR 31-60 ml/min/1,73m^2^ | 1 (9%) |
| GFR 15-30 ml/min/1,73m^2^ | 0 (0%) |
| VAD use | 5 (45%) |
|  |  |
| Adults | 41 |
| Female | 12 (29%) |
| Age (years) | mean 52.4, median 56, range 20-67 |
| Body surface area (BSA) | mean 2.01, median 2.03, range 1.51-2.55 |
| BMI (kg/m^2^) | mean 27.09, median, 26.53, range 19-35.11 |
| Indication for transplant |  |
| Dilated cardiomyopathy | 24 (59%) |
| Ischemic cardiomyopathy | 6 (15%) |
| Other cardiomyopathy | 6 (15%) |
| CHD: biventricular physiology | 4 (10%) |
| CHD: univentricular physiology | 0 (0%) |
| Retransplant | 1 (2%) |
| Kidney function pre-transplant |  |
| GFR ≥ 90 ml/min/1,73m^2^ | 4 (10%) |
| GFR 61-89 ml/min/1,73m^2^ | 11 (27%) |
| GFR 31-60 ml/min/1,73m^2^ | 24 (59%) |
| GFR 15-30 ml/min/1,73m^2^ | 2 (5%) |
| VAD use | 10 (24%) |

CHD: congenital heart disease, GFR = glomerular filtration rate, VAD = ventricular assist device.

Body surface area (BSA) was calculated using Mosteller Method: BSA (m^2^) = (height (cm) x weight (kg)/3600)^½^

Supplemental table 2: donor characteristics

| All patients | 52 |
| --- | --- |
| Donor age | mean 41.33, median 49.5, range 0-73 |
| Donor Body surface area (BSA) | mean 1.82, median 1.86, range 0.41-2.67 |
| Donor BMI (kg/m^2^) | mean 24.6, median 23.8, range 13.7-62.5 |
| Donor heart ischemic time (min) | mean 180.8, median 183.5, range 63-394 |
|  |  |
|  |  |
| Children | 11 |
| Donor age | mean 17.27, median 13, range 0-50 |
| Donor Body surface area (BSA) | mean 1.31, median 1.41, range 0.41-1.85 |
| Donor BMI (kg/m^2^) | mean 18.4, median 17.8, range 13.7-23.9 |
| Donor heart ischemic time (min) | mean 225.6, median 222, range 96-394 |
|  |  |
| Adults | 41 |
| Donor age | mean 47.8, median 53, range 15-73 |
| Donor Body surface area (BSA) | mean 2.0, median 1.9, range 1.6-2.7 |
| Donor BMI (kg/m^2^) | mean 26.3, median 25.2, range 17.2-62.5 |
| Donor heart ischemic time (min) | mean 168.8, median 173, range 63-392 |

Body surface area (BSA) was calculated using Mosteller Method: BSA (m^2^) = (height (cm) x weight (kg)/3600)^½^

Supplemental table 3: induction and maintenance immunosuppression after transplant

|  | Induction | Maintenance |
| --- | --- | --- |
| Adults | High-dose methylprednisolone  ATG | TAC/MMF/Pred, Pred usually tapered by 1 year  Low-dose TAC/EVE/MMF/Pred if pre-existent kidney damage |
| Children | High-dose methylprednisolone  Basiliximab  Plasmapheresis if PRA high or high AB0-titers in case of AB0-incompatibel transplant | TAC/MMF/Pred, Pred usually tapered by 9 months  Low-dose TAC/EVE/MMF/Pred if pre-existent kidney damage |

ATG= anti-thymocyte-globulin, EVE = Everolimus, MMF = mycophenolate mofetil, PRA = panel-reactive antibodies, Pred = Prednisolone, TAC = Tacrolimus

Supplemental table 4: surveillance schedule after transplant

All patients were subjected to regular visits to the transplant center for endomyocardial biopsies (EMB), echocardiography, EKG, physical examination, and labs (blood count, electrolytes, cardiac enzymes, infection parameters, liver and kidney studies, cytomegalovirus and Epstein-Barr-virus load, troughs for immunosuppressants and donor specific antibodies). In our study, blood samples were collected coincident but before EMB were obtained, during the first year after transplant. Additional samples were collected three days after treatment for suspected rejection was started.

| Adults | EMB at weeks 2, 3, 4, 5 or 6 and 8  EMB at months 3, 4, 6, 9 and 12 (9 months is skipped in some uncomplicated cases)  After 1 year, EMB is done only if rejection is suspected | |
| --- | --- | --- |
| Children | Infants | EMB at 6 months or if weight > 6-7 kg  EMB at 12 months  EMB every year until 6 years of age |
|  | > 1 year | EMB at 1, 3, 5 weeks  EMB at 3, 4, 6, 9, 12 months  EMB year 1, 2, 3 after transplant |

Supplemental table 5: treatment for acute rejection

EMB were classified according to the ISHLT classification scheme, revised version from 2004. Acute cellular rejection (ACR) grade 1 was usually not treated, except for grade 1 on the first EMB in highly immunized patients. Grades 2 and 3 were always treated. Antibody-mediated rejection (AMR) was always treated.

|  | ACR | AMR |
| --- | --- | --- |
| Adults | - Without hemodynamic compromise: high-dose methylprednisolone - With hemodynamic compromise: high-dose methylprednisolone or ATG | - ATG. Plasmapheresis or Rituximab as indicated. |
| Children | - high-dose methylprednisolone. ATG if rejection doesn’t resolve | - high-dose methylprednisolone and Rituximab, intravenous immunoglobulins and plasmapheresis as indicated. |

ATG= anti-thymocyte-globulin

Supplemental table 6: Detailed assays and primer/probe sequences

|  | **Name** | **Chr** | **SNP** | **Length of amplicon** | **Forward primer** | **Reverse primer** | **Annealing temperature ˚C** | **Probe A (FAM)**** | **Probe B (HEX)**** |
| --- | --- | --- | --- | --- | --- | --- | --- | --- | --- |
| 1 | kgp2846187 | 16 | C>G | 90 | tccagcagaggaaatagtacttgc | agccacctggtctcctttca | 59 | ctgggagagaaagaacaaaCagcat | catttccccaaatgctCtttgttct |
| 2 | kgp779610 | 8 | C>G | 101 | gtgggcagtctcactggag | cagtgtggctctgctgtgg | 61 | aggCctgggtggagaagt | ccagCccttgtctcaaaagcc |
| 3 | kgp88374 | 8 | C>G | 105 | ggacactcactggggcctct | aggactgaaactagaagaaaaggtcgg | 59 | agacaCttgtgggactcagaagg | acaaCtgtctcctgctgtcct |
| 4 | kgp12502655 | 7 | A>G | 88 | aggcagaactaaacgttggctt | tgcggaacagtgacaatttgttc | 59 | atgcAgctttggcatgaggt | atgccaaagcCgcatattttctct |
| 5 | rs11103106 | 9 | A>G | 98 | cagggagtgctttactgaggca | actcaaacacggagctgggc | 59 | ggcagcaggtgccAagca | aggcattactgctCggcacc |
| 6 | kgp5357482 | 22 | A>C | 89 | tggttgaacgtccacagaagga | caagcacacgtggctgctc | 59 | cagTgccctctgccaggaa | gggcCctgcctgagcatag |
| 7 | kgp9771053 | 18 | A>G | 83 | agccctgcacactcacttacc | tggcattcagatcatcaggcttct | 59 | ccatcaggtgctggcActc | tgcagggaagagCgccag |
| 8 | kgp4246032 | 9 | T>C | 76 | cttcccttgcccctcttcca | tgctctgtggatccctggag | 59 | aggttgtgtgaaAgtgccct | agccctcagggcacCttca |
| 9 | GA002729 | 2 | C>G | 100 | gtctctgggggtctgttggcc | agaggaaggactcccaggggg | 61 | tggagacgggtccgCagag | tggcacaggtgctctCcgg |
| 10 | kgp3586059 | 10 | C>G | 90 | tcaatcctcacaacttccctaaggg | agtgggagggaggtacagtga | 61 | aaaagggggtggtgtCaatgtc | agggactgacattCacaccacc |
| 11 | kgp3469073 | 16 | C>G | 91 | gatcaactcctgaagagactccgt | agggagggatggagagggac | 61 | cgggagccctgcgCtttg | tttccatgacaaaCcgcaggg |
| 12 | kgp5728993 | 1 | C>G | 91 | tcttgtcgaggctgccctgaaagg | acagagccggccggtcgc | 61 | cggttttcgctcCcgtgaa | agtccatttcacgCgagcg |
| 13 | kgp7257211 | 15 | C>G | 106 | gcactatgcttgagtctgtg | atttagatatgtgtgtttttgcctt | 61 | ctttagctgccaagaaggatCagag | agaatgtgtgttctcactctCatcct |
| 14 | kgp9738136 | 18 | C>G | 98 | tggttaaactgtagtacatccatgga | accttttgggactggctttct | 61 | acttctcagcaacagCctgga | ctctggaaattcatccagCctgt |
| 15 | kgp12078903 | 8 | C>G | 92 | agaaagaaagaagcagggaagggac | tggagctaaaatgagcctgcgt | 61 | attacatagcttatcaCttgcagagcc | actcctggctctgcaaCtgat |
| 16 | kgp5942754 | 7 | C>G | 103 | gctgttgctgcctcacaggt | agggcaaaggcaaatgcacca | 61 | aactggaagtaacacCtgcacca | cttgactcttggtgcaCgtgt |
| 17 | kgp7251638 | 11 | C>G | 98 | accctgaccctcagttcctt | aagagcccttataaggtgtgagaaa | 61 | aggatattgctagagtggagtCagaac | accactgttatttgttctCactccact |
| 18 | kgp7882745 | 19 | C>G | 86 | atgaagagtaagcggggccg | cggacccatttcacccacca | 61 | cccgacccttaacCtcccc | tggagagggttggggaCgtta |
| 19 | rs6436409 | 2 | C>G | 97 | tggcccagttagaaggtgtgga | cggccacccatcctggagat | 61 | accctcctgtactgCgcac | acagtgaaggtgtgcCcagt |
| 20 | rs2072042 | 16 | T>C | 96 | gggcctcagttctagacgagt | gtttccgtgaagtaggcgct | 61 | atgctcagcacacAgggga | cactgcttccccCgtgtg |
| 21 | kgp3747074 | 6 | A>G | 96 | aacttagctgctcttgcttcagt | gtacctgccttaactcagtatgatctt | 61 | cccagcaggaaagcgAgtc | aagtaagaatcagacCcgctttcc |
| 22 | rs10228737 | 7 | T>C | 79 | tttgcacttgacgcaccagc | ccgaggcagaggaaggaagtg | 61 | tgcAatgagagcagaggcct | catCgcagccctcctgca |
| 23 | rs13317873 | 3 | T>C | 116 | ggttttgcttctgatgatccctct | agcattgtgtagggactggtaaatt | 61 | atacActctgttgttgagtgccac | cagagCgtatgtatgaagtccagagt |
| 24 | rs10164176 | 18 | T>C | 96 | ccccaaactaagtacctaatcactcgt | ccaaggggagcatccaccat | 61 | cccaCgggaggaatgtctttg | cccAtgggacttctggcc |
| 25 | rs251022 | 5 | A>G | 94 | acacacacacacgcaattcgg | atgagctgaggtgggtgctg | 61 | acacaAagtggcctcccg | acaGagtggcctcccgat |
| 26 | rs10734083 | 10 | T>C | 94 | ggcatctgaattcaagctttggtc | ttcttctagttggtctggtaggct | 61 | aggcttgtacactCtccccc | acactgggatgggggaAagt |
| 27 | kgp187715 | 19 | C>G | 95 | tggttattgttactaggtccccacc | agaataagcaagatgttggcagtgag | 61 | aggactttattggggaggCtgac | ctggaagccaaagtcaCcctc |
| 28 | kgp5971873 | 5 | A>G | 107 | agcttcagcttagacagatgtttattt | cactatcatctccatttcacagacc | 61 | ccCgcagttgcacagcttg | actgcAggccacaaggtg |
| 29 | rs7072759 | 10 | A>G | 83 | ctggggcagagtggagagtc | atccacctctgaacccagcc | 61 | aggacActgcagctgtgg | cagCgtcctctgtgctacct |
| 30 | kgp5873854 | 15 | T>C | 81 | tcccaggctccaggtcagat | ggatcaatgtggctgctccct | 61 | tctcCgcccttctgagatgc | agggcAgagactctggaact |
| 31 | rs12064796 | 1 | A>G | 96 | ggcaaagtgggcaagggtct | gcctcctaaagcttgagccaca | 61 | ttggggccaGgtacctgg | tggggccaAgtacctggt |
| 32 | rs4632826 | 5 | T>C | 143 | agctttcttgcttctgcccca | gggtgccattgcccagagat | 61 | ccctggggccatcaGgtt | ccctggggccatcaAgttt |
| 33 | rs1265094 | 6 | A>G | 96 | accccaagaggctttatagggg | ccttcccaacgggtttgacc | 61 | ccactgggctggCccctc | agtggaggagggAccagc |
| 34 | rs11610836 | 12 | T>C | 96 | acactcctgctgcgtgtctg | ttcctccccaccactcccat | 61 | ggtcccagctggtCgtgg | atgctccccacAaccagct |
| TSPY1* |  | Y |  |  |  |  | 60 |  |  |

Primer 1-34: Primer sequences from Beck et al. In primer number 2 & 28, the primer sequence was changed

*: manufactured by TATAA Biocenter (TATAA Biocenter AB, Gothenburg, Sweden)

**: the polymorphic base is indicated by an upper letter

Chr = chromosome, SNP = single nucleotide polymorphism

Supplemental table 7: dPCR-results for the excluded samples

|  | DF | dd-cfDNA |
| --- | --- | --- |
| n | 66 | 66 |
| Minimum | 0.0125 | 5.145 |
| 25% percentile | 0.1933 | 33.94 |
| Median | 0.3225 | 48.74 |
| 75% percentile | 0.4575 | 88.94 |
| Maximum | 5.250 | 352.3 |
|  |  |  |

DF is expressed as percent, dd-cfDNA in copies/ ml
