# Supplementary material for: Absolute Quantification of Donor-Derived Cell-Free DNA in Pediatric and Adult Patients After Heart Transplantation: A Prospective Study
Source: Transpl Int. 2023 Oct 30;36:11260. doi: 10.3389/ti.2023.11260 (PMC10641041; doi:10.3389/ti.2023.11260)
Supplement: Supplementary file 2 [file DataSheet1.docx]

**Supplemental Fig. 1:** **Summary of the workflow**

HTx = heart transplantation, cfDNA = cell-free DNA, rd-cfDNA = recipient-derived cfDNA, dd-cfDNA = donor-derived cfDNA, DF= donor fraction, dPCR = droplet digital PCR, PA = preamplification, SNP = single nucleotide polymorphism, Y = Y-chromosome.
